# Physiological Effects of Superoxide Dismutase on Altered Visual Function of Retinal Ganglion Cells in db/db Mice

**DOI:** 10.1371/journal.pone.0030343

**Published:** 2012-01-17

**Authors:** Chunxia Xiao, Meihua He, Yan Nan, Dongjuan Zhang, Baiyu Chen, Youfei Guan, Mingliang Pu

**Affiliations:** 1 Department of Anatomy, School of Basic Medical Sciences, Peking University, Beijing, China; 2 Key Laboratory on Machine Perception, Peking University, Beijing, China; 3 Key Laboratory for Visual Impairment and Restore, Peking University, Beijing, China; 4 Department of Physiology and Pathophysiology, School of Basic Medical Sciences, Peking University, Beijing, China; Institut de la Vision, France

## Abstract

**Background:**

The C57BLKS/J db/db (db/db) mouse is a widely used type 2 diabetic animal model, and this model develops early inner retinal neuronal dysfunction beginning at 24 weeks. The neural mechanisms that mediate early stage retinal dysfunction in this model are unknown. We evaluated visual response properties of retinal ganglion cells (RGCs) during the early stage of diabetic insult (8, 12, and 20 wk) in db/db mice and determined if increased oxidative stress plays a role in impaired visual functions of RGCs in 20 wk old db/db mice.

**Methodology/Principal Findings:**

*In vitro* extracellular single-unit recordings from RGCs in wholemount retinas were performed. The receptive field size, luminance threshold, and contrast gain of the RGCs were investigated. Although ON- and OFF-RGCs showed a different time course of RF size reduction, by 20 wk, the RF of ON- and OFF-RGCs were similarly affected. The LT of ON-RGCs was significantly elevated in 12 and 20 wk db/db mice compared to the LT of OFF-RGCs. The diabetic injury also affected contrast gains of ON- and OFF-RGCs differently. The generation of reactive oxidative species (ROS) in fresh retina was estimated by dihydroethidium. Superoxide dismutase (SOD) (300 unit/ml) was applied in Ames medium to the retina, and visual responses of RGCs were recorded for five hours. ROS generation in the retinas of db/db mice increased at 8wk and continued to progress at 20 wk of ages. *In vitro* application of SOD improved visual functions in 20 wk db/db mice but the SOD treatment affected ON- and OFF-RGCs differently in db/m retina.

**Conclusions/Significance:**

The altered visual functions of RGCs were characterized by the reduced RF center size, elevated LT, and attenuated contrast gain in 12 and 20 wk db/db mice, respectively. These altered visual functions could, at least partly, be due to oxidative stress since *in vitro* application of SOD effectively improves visual functions.

## Introduction

Diabetic retinopathy (DR) is classically thought of as a microvascular disease of the retinal capillaries. However, diabetes also impairs retinal neuronal function before the onset of visible vascular lesions. For example, numerous visual psychophysical and electrophysiological investigations reveal that type 2 diabetes mellitus patients show visual function defects without retinopathy [1]–[4]. Furthermore, impaired contrast sensitivity is recognized as an early sign of neural retinal dysfunction [5]–[8]. In addition, different types of retinal neuronal deficits have been reported in diabetic animal models prior to the onset of vascular compromise including alterations of photoreceptor [9]–[12], bipolar [13], [14], amacrine [15], [16] and ganglion cell [17]–[20] function. However neural mechanisms that mediate early stage diabetic retinal dysfunction remain to be determined.

One of the unique characteristics of retinal neurocircuitry is its parallel ON and OFF signal pathways. There is growing body of evidence that the ON pathway can be preferentially affected by diabetes (for review see [21]). Indeed, physiological recordings from rod driven ON-bipolar cells also show that the GABA signaling pathway is altered in diabetic retinas [22]. Morphological studies report that a significant enlargement of RGC dendritic fields of diabetic rats [23] and an increase in dendritic branching in ON type RGCs of Ins2akita/+ mice [24]. Most of these results are collected from type I diabetic animal models and to date retinal neural structural and functional studies of type II diabetic animal model are scarce. The C57BLKS/J db/db (db/db) mouse is a popular genetic model of type 2 diabetes. It develops hyperglycemia starting at 8 wk of age [25]. The persistent hyperglycemia results in reduced thickness in photoreceptor layer, inner nuclear layer (INL), and ganglion cell layer (GCL) at 14 wk of age [26]. Physiological studies have revealed early inner retinal neuronal dysfunction includes prolonged latencies of the oscillatory potentials and an impaired b-wave at 24 wk of age [27]. An increased apoptosis of RGCs and other retinal neurons was observed in 60 wk old db/db mice [28]. However, the structural and functional properties of RGCs in db/db mice during the early stage of diabetes are unknown.

Oxidative stress, induced by increased accumulation of ROS and/or decreased antioxidant capacity, plays an important role in the pathogenesis of DR [29], [30]. Increased generation of ROS avidly interacts with a large number of molecules including other small inorganic molecules as well as proteins, lipids, carbohydrates, and nucleic acids. Through such interactions, ROS may irreversibly destroy or alter the function of the target molecule [31]. Oxidative stress is closely related to the vascular changes in diabetic retinopathy. Increased ROS generation in diabetic retina has been confirmed by several groups [28], [32]. However, little is known about how ROS might affect primary visual functions of RGCs such as receptive field size, luminance threshold, and contrast gain during the early stage of DR. Multiple lines of evidence show that treatments targeting formation of ROS and peroxynitrite exert neuroprotective effects in vitro and in vivo [33]–[38]. Superoxide dismutase (SOD) is an important antioxidant defense in almost all living cells. There is a wealth of literature demonstrating that SOD plays a pivotal role in protecting injured RGCs in variety of animal models of retinal disease [39]–[42]. Furthermore, decreased activity and expression of SOD has also been found in the diabetic retina [43], [44]. However, no research to date has adequately tested if SOD could improve altered visual functions of RGCs during early stages of diabetic retinas in db/db mice.

Based on these considerations, the goal of present study was three-fold: 1) evaluate visual response properties of retinal ganglion cells during the early stage of diabetic injury; 2) evaluate visual response properties of ON- and OFF-RGCs in db/db and db/m mice in different age groups; 3) investigate if oxidative stress plays an role in stressed visual functions of ON- and OFF-RGCs in 20 wk db/db mice.

## Results

Since db/db mice develop hyperglycemia starting at 8 wk of age [7], [8], the animals were divided into three age groups, 8, 12, and 20 wk, respectively. Animal body weight and glucose level were summarized in Table 1. We started this investigation by evaluating general visual response properties of RGCs including receptive field size, luminance threshold, and contrast gain first. Then visual response properties of ON- and OFF-RGCs were analyzed. It was followed by detecting intracellular ROS levels in db/db and db/m mice retina, and finally, we evaluated the effect of SOD on the visual functions of RGCs in 20 wk db/db mice.

**Table 1:** Animal body weight and glucose level.

Age	N	Body weight (g)	Blood glucose (mmol/l)
**8** **wk**			
db/m	11	18.6±0.7	7.5±0.2
db/db	15	32.7±1.1***	9.1±0.4*
**12** **wk**			
db/m	10	19.6±0.5	7.8±0.2
db/db	10	42.7±1.6***	26.1±1.3***
**20** **wk**			
db/m	20	24.1±0.4	8.3±0.2
db/db	20	52.8±1.2***	27.6±0.7***

Values are means±SEM.

***p<0.0001

*p<0.05 vs dm.

### Receptive field center size

The RF center was mapped with a 0.2° testing spot. An area-threshold test was then performed to determine which spot size evoked the maximum discharge. This size was defined as the RF size of the recorded RGC. Table 2 summarizes RF center size data collected from db/db and its age-matched litter mate db/m mice. A total of 84 RGCs (ON = 39: 8 wk = 14, 12 wk = 15, 20 wk = 10 and OFF = 45: 8 wk = 21, 12 wk = 12, 20 wk = 12) from db/m mice and 116 RGCs from db/db mice (ON = 40: 8 wk = 16, 12 wk = 13, 20 wk = 11 and OFF = 71: 8 wk = 32, 12 wk = 21, 20 wk = 18) were recorded. We analyzed ON and OFF RF diameter changes with two-way ANOVA analysis.

**Table 2:** Average receptive field diameter of retinal ganglion cells in db/m and db/db mice.

Age	Number of recorded cells		Average RF diameter (deg)	
	ON	OFF	ON	OFF
**8** **wk**				
db/m	14	16	27.3±3.0	31.5±2.9
db/db	21	32	27.2±2.7	26.7±2.1
**12** **wk**				
db/m	15	13	31.0±2.9	30.0±3.5
db/db	12	21	19.3±1.3*	25.9±2.2
**20** **wk**				
db/m	10	11	27.2±3.8	33.0±3.7
db/db	12	18	16.9±1.0*	21.2±0.7**

Values are means±SEM.

**P<0.001

*p<0.05 vs dm.

**Figure 1:**
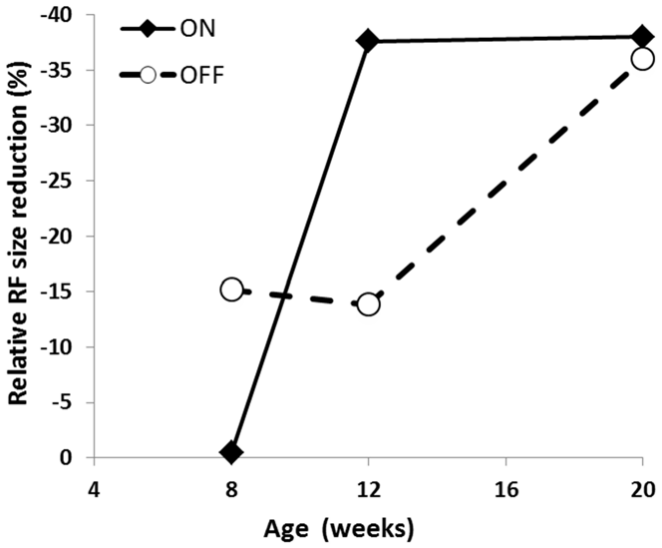
Receptive field size reduction in ON- and OFF-RGCs. The abscissa is animal age (wk) and vertical coordinate depicts relative percentage of RF size reduction. Filled diamond and solid line represents ON-RGC while open circle and dashed line shows OFF-RGC RF size reduction.

As shown in Table 2, there were significant reductions in the RF size of ON-RGCs from 12 wk (p<0.004, two-way ANOVA) and 20 wk db/db mice (p<0.02, two-way ANOVA), respectively. Similarly, there was a significant OFF-RGC RF size shrinkage in 20 wk db/db animals (p<0.005, two-way ANOVA). Furthermore, the RF size of OFF-RGCs was significantly larger than that of ON-RGCs from 12 wk (p<0.04, one-way ANOVA)) and 20 wk (p<0.002, one-way ANOVA) db/db mice, respectively. Then, we compared relative RF size reductions of db/db to db/dm mice at three time points are: 8 wk: ON 100%, OFF 85%; 12 wk: ON 62%, OFF 86%; 20v wk: ON 62%, OFF 64%. The OFF system had a consistent ∼15% reduction in relative RF size from the first two time points (8–12 wk) whereas the ON system showed no reduction at the first time point but a substantial shrinkage at the next two time points (12–20 wk). Thus, as shown in Figure 1, ON and OFF systems showed different trends of RF size reductions but both systems were affected similarly at the last time point (20 wk) (ON: 62%; OFF: 64%).

### Luminance threshold

**Figure 2:**
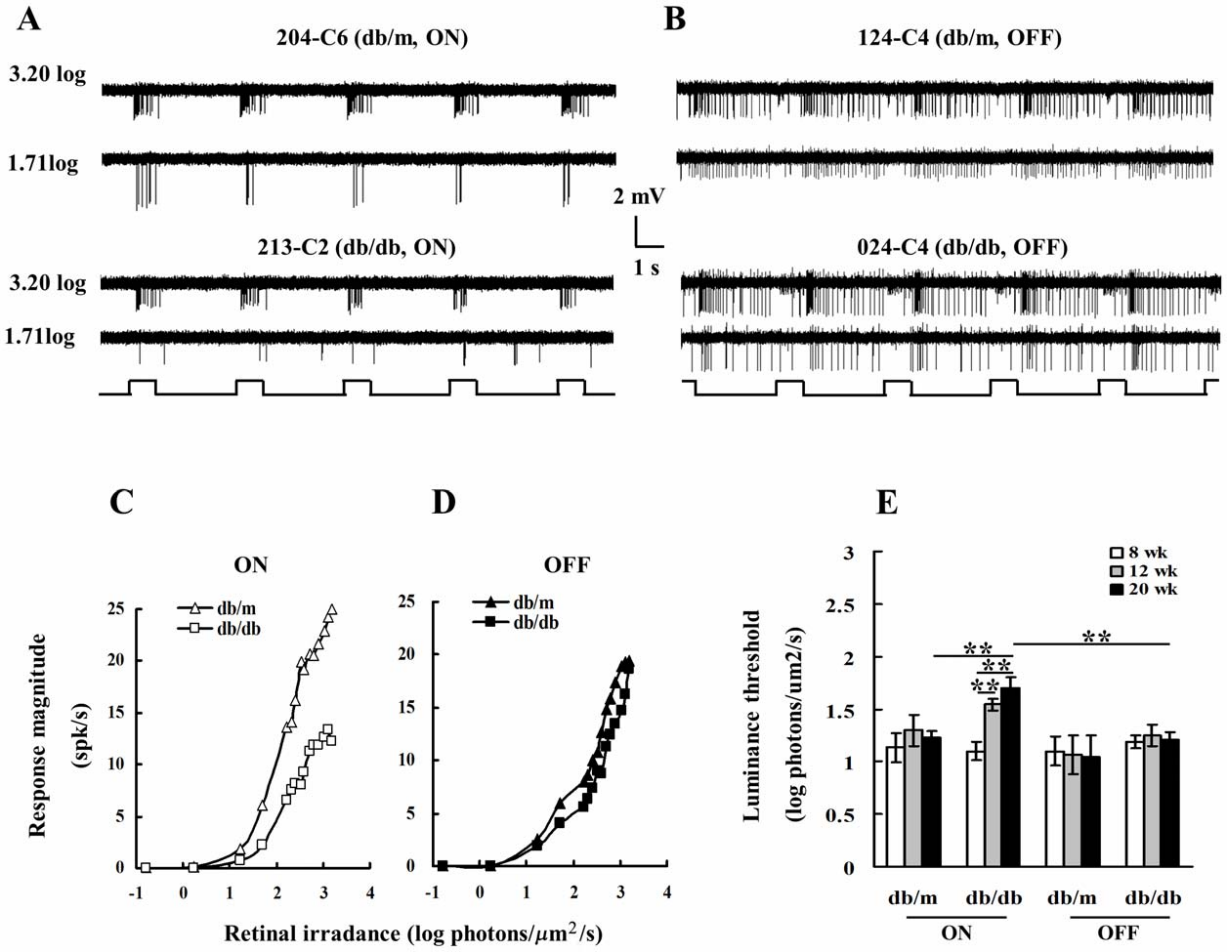
Luminance response patterns of RGCs recorded from 20 wk old db/db and db/m retinas. **Panel A:** Discharge patterns of two ON-center RGCs. Each cell responded to a light spot centered on the RF. The top two traces exhibit discharge patterns of an ON-RGC (204-C6) recorded from a db/m retina (eccentricity: 0.98 mm) and bottom two traces show discharge patterns of an ON-RGC (213-C2) recorded from a db/db retina (eccentricity: 1.6 mm). Irradiance levels (log photons/µm^2^/s) are shown to the left of the response traces. The spot was turned on for 1 sec and the duty circle was 3 sec. The unattenuated background irradiance level was 5.5 photons/µm^2^/s. **Panel B:** Discharge patterns of two OFF-center RGCs. The top two traces exhibit discharge patterns of an OFF-RGC (124-C4) from a db/m retina (eccentricity: 1.46 mm) and bottom two traces show discharge patterns of an OFF-RGC (024-C4) from a db/db retina (eccentricity: 1.78 mm). All stimulus convention as Panel A. **Panel C:** Luminance response profiles of the two ON cells exhibited in Panel A at various irradiance levels. The abscissa is retinal irradiance and vertical coordinate depicts response magnitude (spk/sec). The open triangles depict db/m ON; open squares represent db/db ON. **Panel D:** compares luminance response profiles of two OFF-RGCs illustrated in Panel B: filled triangles: db/m OFF; and filled squares: db/db OFF. **Panel E:** Histogram comparing the average threshold irradiance of ON- and OFF-RGCs within each group. Data represent the mean±SEM of 8 cells, *P<0.05; **P<0.001; *** P<0.0001, one-way ANOVA with Tukey's multiple comparison test or two-way ANOVA followed with post hoc analysis.

Since both ON- and OFF-RGCs showed significantly reduced RF size in 20 wk db/db mice, we anticipated a progressive depression of luminance sensitivity. As shown in Figure 2, ON- and OFF-RGCs showed different luminance response patterns. Figure 2 compares luminance responses between ON- and OFF-RGCs of 20 wk old db/db and db/m mice at retinal irradiance levels of 3.20 and 1.71log photons/µm**^2^**/s, respectively. Panel A depicts the luminance responses of two ON center RGCs, one of them was recorded from db/m (204-C6, top left) and another from db/db retina (213-C2, bottom left). Both cells responded well to 3.2 log photons/µm**^2^**/s, however as irradiance fell to 1.71, the latter response reduced substantially. Panel B compares luminance responses of two OFF center RGCs. The cell (124-C4, top right) from db/m retina exhibited vigorous discharges at both irradiance levels. The cell (024-C4, bottom right) from db/db retina showed a well-modulated OFF response pattern at 3.2, as the visual stimulus irradiance went down to 1.71, the discharge frequency was reduced. Panel C reveals the luminance response profiles of the two ON-RGCs exhibited in Panel A under different retinal irradiance levels. The response curve of 213-C2 moved down vertically indicating considerably attenuated luminance responses. Panel D represents luminance response curves of the two OFF-RGCs exhibited Panel B. As shown in the panel, in comparison with of 124-C4, the response curve of 024-C4 moved down slightly, suggesting the latter had a minor decline in luminance response. This response pattern was confirmed in the majority of recorded cells. Panel E compares LTs of ON- and OFF-RGCs recorded from db/m and db/db retinas. As shown in the histogram, there was no statistical difference between the three age groups of db/m mice in either ON- or OFF-RGCs (p>0.05, one-way ANOVA). The same was true for OFF-RGCs recorded from db/db mice. Nevertheless, in comparison with 8 wk ON-RGCs, 12 and 20 wk ON-RGCs of db/db mice showed a significantly elevated LT (8–12 wk: ***p***<0.01; 8–20 wk: ***p***<0.001, one-way ANOVA). Consequently, there was a significant difference in LT between the ON-RGCs recorded from 20wk db/m and db/db mice (***p***<0.0006, two-way ANOVA) as well as between the ON-RGCs of 20wk db/db and the OFF-RGCs of 20 wk db/db (***p***<0.001, one-way ANOVA).

### Contrast gain

**Figure 3:**
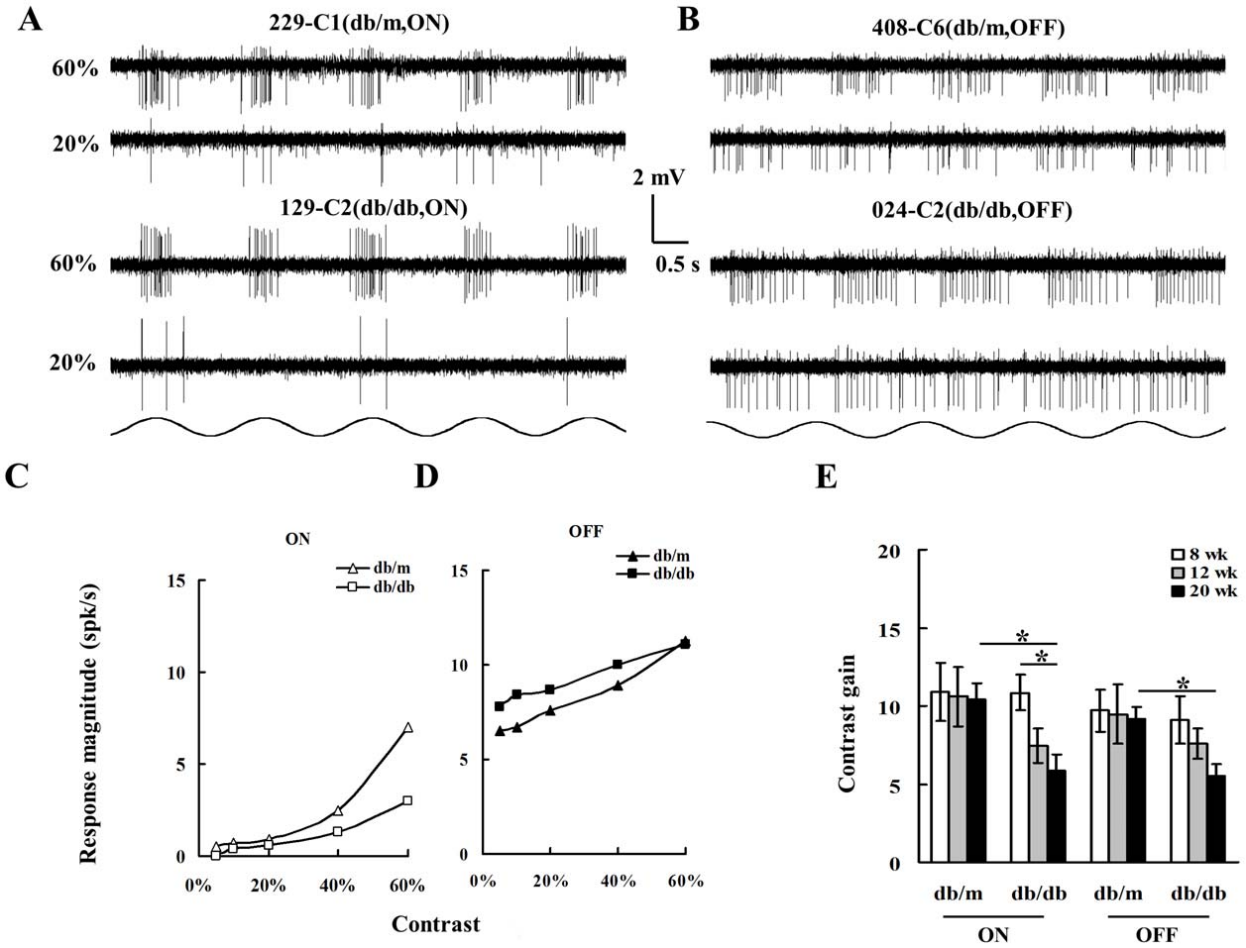
Contrast responses of RGCs recorded from 20 wk old db/m and db/db retinas. **Panel A:** Discharge patterns of two ON-center RGCs in response to 0.02 cyc/deg sinusoidal drifting gratings at two different contrast levels, 60% and 20%, respectively. The top two traces exhibit discharge patterns of an ON-RGC (229-C1, top left) recorded from a db/m retina (eccentricity: 1.27 mm) and bottom two traces show discharge patterns of an ON-RGC (129-C2, bottom left) recorded from a db/db retina (eccentricity: 1.05 mm). Contrasts are shown to the left of the response traces. **Panel B:** Discharge patterns of two OFF-RGCs. The top two traces exhibit discharge patterns of an OFF-RGC (408-C6, top right) recorded from a db/m retina (eccentricity: 0.95 mm) and the bottom two traces show discharge patterns of an OFF-RGC (024-C2) recorded from a db/db retina (eccentricity: 1.41 mm). **Panel C:** contrast gain of the two ON cells exhibited in Panel A. The abscissa is the contrast and vertical coordinate depicts contrast response magnitude (spk/sec). The open triangles depict db/m ON; open squares represent db/db ON. **Panel D:** compares the contrast gain of two OFF-RGCs illustrated in Panel B: filled triangles: db/m OFF; and filled squares: db/db OFF. Panel E: Histogram comparing average contrast gains of ON and OFF RGCs of within each group. Other conventions are as for Figure 2.

Figure 3 illustrates contrast responses between RGCs of 20 wk db/db and db/m at 60% and 20% contrast, respectively. Panel A shows two ON-RGCs, cell 229-C1 (top left) was recorded from a db/m mouse and 129-C2 (bottom left) from a db/db mouse, in response to the sinusoid gratings (0.02 c/deg) drifted across the receptive field center at two contrast levels (60% and 20%), respectively. Both cells responded equally well to 60% contrast gratings. As the contrast level reduced to 20%, cell 429-C1 showed reduced but consistent discharges, while the cell 129-C2 from the db/db retina only responded to three out of five stimuli. Panel B compares the contrast responses of two OFF-RGCs; Cell 408-C6 of db/m retina (top right) had a vigorously firing pattern at 60% contrast and continued to respond at 20%. The cell 024-C2 from db/db retina (bottom right) showed a well-modulated discharge pattern at 60% contrast, and it was still responsive as the contrast leveled down to 20%. Panel C depicts contrast response profiles of the two ON-RGCs cells described in Panel A. The contrast gain was calculated to evaluate contrast sensitivity of recorded cells. The contrast gains were 11.26 and 5.04 for 229-C1 and 129-C1, respectively. Panel D displays contrast response profiles for the two OFF-RGCs illustrated in Panel B. Unlike ON-RGCs though, both OFF-RGCs had higher discharge rate (from ∼6 spk/sec to 11 spk/sec) and shared similar shaped contrast response curves but a substantially different contrast gains, 8.52 and 5.72 for cell 408-C6 and 024-C2, respectively. Panel E depicts the average contrast gains of ON- and OFF-RGCs from db/m and db/db mice at different age groups. The ON-RGCs of 20 wk db/db mice demonstrated contrast gains which were significantly lower than those from the 8 wk group (8–20 wk: ***p***<0.01; One-way ANOVA). Additionally, ON-RGCs recorded from 20 wk db/db mice showed significantly less contrast gain than those from the age-matched db/m mice (***p***<0.04, two-way ANOVA). The average contrast gain of OFF-RGCs in 20 wk db/db mice was significantly lower than that of OFF-RGCs from age-matched db/m mice (p<0.01, one-way ANOVA).

### Measurement of ROS levels in retinas of db/db and db/m mice

**Figure 4:**
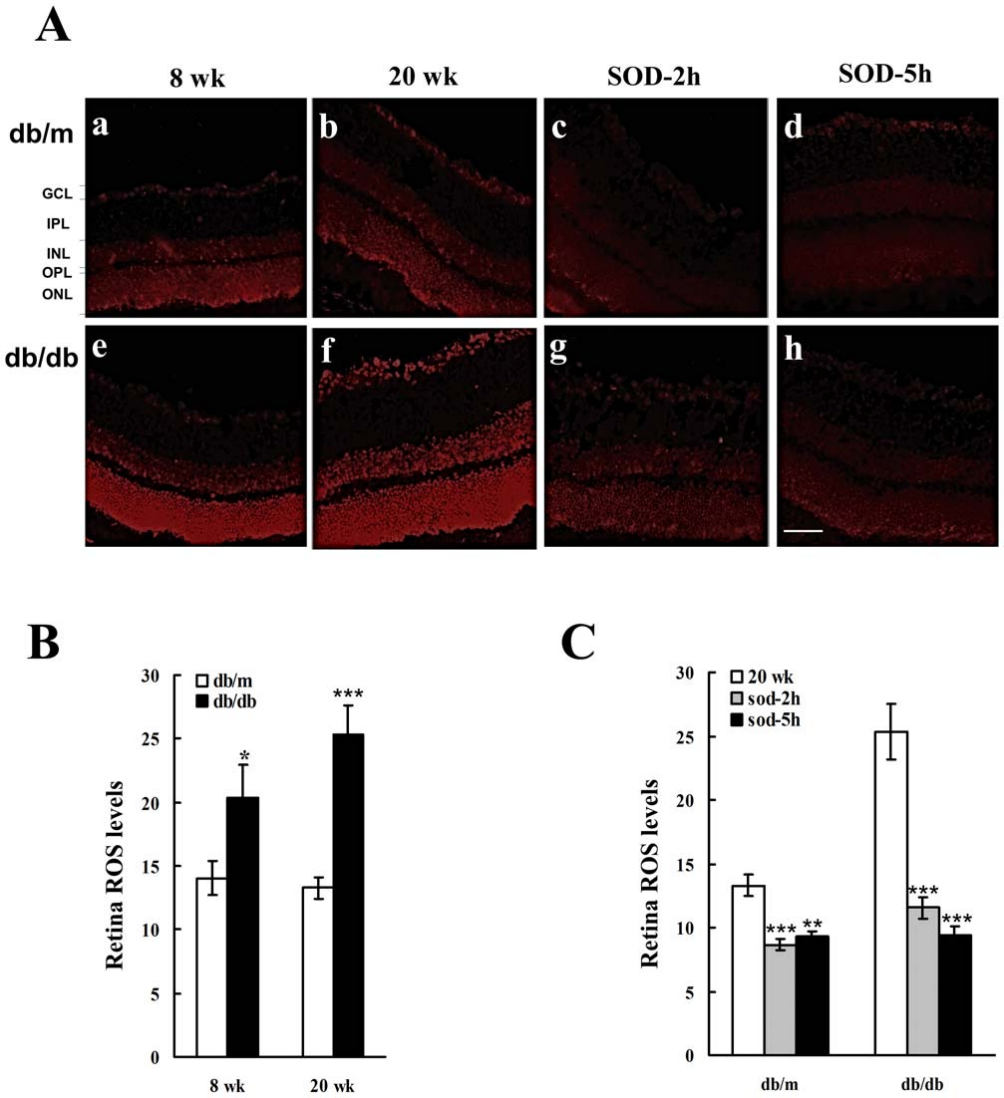
ROS levels of retinal neurons before and after SOD treatment in 8 and 20 wk db/m and db/db mice. In situ ROS generation was determined in freshly prepared retinal sections by immunohistochemical staining for dihydroethidium (DHE). The quantitatively analyze was performed by using an image analysis software (Adobe Photoshop CS5, CA). The thickness of each retinal section was 10 µm. Micrographs of retinal sections that were stained with DHE: **Panel A:** a: 8 wk db/m; b: 20 wk db/m; c: 20 wk db/m, 2-hour SOD treatment; d: 20 wk db/m 5-hour SOD treatment; e: 8 wk db/db; f: 20 wk db/db; g: 20 wk db/db, 2-hour SOD treatment; h: 20 wk db/db, 5-hour SOD treatment. ONL: outer nucleus layer; OPL: outer plexiform layer; INL: inner nucleus layer; GCL: ganglion cell layer. **Panel B:** quantitative analysis of retinal ROS levels. The fluorescent intensity of DHE labeled neurons was quantified (means±SEM, n = 4). In comparison with db/m mice, the intracellular ROS generation was significantly increased in retinas of 8 and 20 wk db/db mice (8 wk: *p<0.05; 20 wk: ***p<0.0001, one-way ANOVA). **Panel C:** SOD treatment for 2- and 5- hour significantly reduced the ROS levels in 20 wk db/db mice retina (2 hours: **p<0.001; 5 hours: ***p<0.0001, one-way ANOVA). Scale bar: 20 µm. Other conventions are as for Figures 2 and 3.

To evaluate the retinal oxidative stress levels in db/db and db/m mice, ***in situ*** ROS generation was measured in freshly prepared retinal sections by DHE. As shown in Figure 4, in comparison with 8 and 20 wk db/m mice (Panel A: a and b), the db/db mice retinas had a significantly elevated ROS generation at 8 wk of age (Panel A: e), and the oxidative stress continued to progress at 20 wk (Panel A: f). Quantitative analysis shows that both 8 and 20 wk db/db mice had significantly higher levels of ROS expression in the retina than db/m mice (Panel B). Retinas from 20 wk db/m and db/db mice were treated with SOD for 3 or 5 hours and reduced ROS levels were observed in both db/m (Panel A: c, d) and db/db retinas (Panel A: g, h). Further analysis shows that SOD application significantly suppressed retinal ROS expression levels in both db/m and db/db mice (Panel C). Therefore it is plausible that the reduced ROS level could relief the oxidative stress and consequently alters the declined visual functions of RGCs in db/db mice. To test this hypothesis we performed following experiment.

### In vitro application of SOD

**Figure 5:**
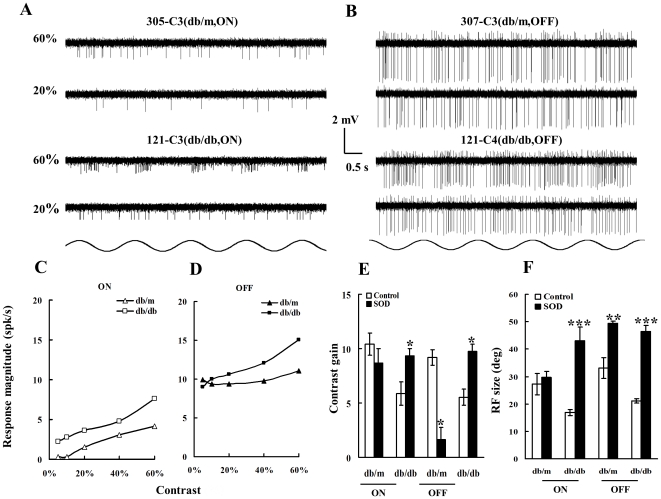
Visual response properties of ON- and OFF-RGCs of 20 wk db/db mice after in vitro SOD application. **Panel A:** Discharge patterns of two ON-center RGCs in response to 0.02 cyc/deg sinusoidal drifting gratings of different contrast levels. The top two traces exhibit discharge patterns of an ON-RGC (305-C3, top left) recorded from a db/m retina (eccentricity: 0.94 mm) and bottom two traces show discharge patterns of an ON-RGC (121-C3, bottom left) recorded from a db/db retina (eccentricity: 1.17 mm). Contrast levels are shown to the left of the response traces. **Panel B:** Discharge patterns of two OFF-RGCs. The top two traces exhibit discharge patterns of an OFF-RGC (307-C3, top right) recorded from a db/m retina (eccentricity: 1.71 mm) and bottom two traces show discharge patterns of an OFF-RGC (121-C4) recorded from a db/db retina (eccentricity: 1.21 mm). **Panel C:** contrast response profiles of the two ON cells exhibited in Panel A. The abscissa is contrast (%) and vertical coordinate depicts contrast response magnitude (spk/sec). The open triangles depict db/m ON; open squares represent db/db ON. **Panel D:** The contrast response profiles of two OFF-RGCs illustrated in Panel B. filled triangles: db/m OFF; and filled squares: db/db OFF. **Panel E:** Histogram comparing average contrast sensitivity of ON and OFF RGCs of within each group. For db/db mice, as a comparison was made between the control (without SOD treatment) and ON-RGCs that received SOD treatment, the average contrast gain of latter was significantly increased (p<0.05, n = 6). As a comparison was made between the control and OFF-RGCs that received SOD treatment, the average contrast sensitivity of latter was significantly improved (p<0.01, n = 6). For db/m mice, after the SOD treatment, the contrast gain of ON-RGCs was not significantly changed whereas that of OFF-RGCs was significantly suppressed (p<0.01, n = 7). **Panel F:** Histogram comparing the average RF size of RGCs after SOD treatment. Other conventions are as for Figure 4.

To verify if increased oxidative stress plays a role in the impaired visual functions of retinas from 20 wk db/db mice, physiological recordings were carried out after the retinas were perfused in oxygenated Ames Medium containing 300 unit/ml of SOD, a superoxide scavenger, for 60 min. Stable SOD activity under room temperature can only last for six hours (Sigma-Aldrich S5395 datasheet), eliminating the possibility for the time intensive luminance threshold measurement and complete dark adaptation. We, therefore, decided to focus on examining RF size and the contrast gain of RGCs after SOD treatment. Physiological recordings were performed after the retinas were perfused in oxygenated Ames Medium that contained 300 unit/ml SOD for 60 min. As shown in Figure 5, Panel A compares two ON-RGCs in response to gratings of different contrasts , one from db/m retina (305-C3, top left) and another from db/db retina (121-C3, bottom left), respectively. The former had weak responses to 60% contrast sinusoidal drifting gratings and the responses diminished at 20%. The latter from db/db retina, however, not only exhibited vigorous discharge patterns at the contrast of 60% but also responded well to gratings at 20% contrast. This is the behavior that one would expect from a db/m retina. Panel B shows two OFF-RGCs: one from 20 wk db/m retina (307-C3, upper left) and another from 20 wk db/db retina (121-C4, bottom left). The cell 307-C3 had weak discharge patterns at 60% and did not respond to 20% contrast. In contrast, the cell recorded from diabetic retina (121-C4) had well-modulated responses to gratings of both 60% and 20% contrasts. Panel C illustrates the contrast response profiles of the two ON-RGCs exhibited on Panel A, while Panel D does the same for the two OFF-RGCs illustrated in Panel B. Panel C shows that, after SOD treatment, the ON-RGC from the db/db retina had a contrast gain of 9.23 while it was 7.45 for the cell from db/m retina, while panel D does the same for the two OFF-RGCs illustrated in Panel B. The OFF-RGC from db/db retina had a contrast gain of 10.26 while the gain was 2.42 for the OFF-RGC from db/m retina. Thus both ON and OFF RGCs of db/db mice performed better than their counterparts in db/m mice after SOD treatment. Pane E summarizes the contrast gain of ON-RGCs (db/m: n = 6; db/db: n = 6) and OFF-RGCs (db/m: n = 12; db/b: n = 6) after the SOD treatment. As shown in the histogram, in comparison with RGCs from 20 wk db/db retinas, the contrast gains of ON- and OFF-RGCs were significantly enhanced after the treatment (ON: p<0.05, OFF: p<0.01, one-way ANOVA). However, the impact of SOD on ON- and OFF-RGCs of db/m mice was different. It had little affect on contrast gain of ON-RGCs (p>0.05, one-way ANOVA) but significantly suppressed that of OFF-RGCs (p<0.01, one-way ANOVA). Panel F shows RF size changes in response to SOD treatment. The histogram illustrates a significantly increased RF size for both ON- (p<0.00005, one-way ANOVA) and OFF-RGCs (p<0.00001, one-way ANOVA) in db/db mice after SOD treatment. However, ON- and OFF-RGCs of db/m mice had different response patterns to the treatment. Similar to their counterparts in db/db animals, OFF-RGCs had significantly increased RF size (p<0.0002, one-way ANOVA). Nevertheless, there was no significant change to the RF size of ON-RGCs (p>0.05, one-way ANOVA).

## Discussion

This study demonstrates that the declination of the visual response properties of RGCs, including RF size, luminance response, and contrast gain in db/db mice. The application of a ROS scavenger, SOD, quickly improved impaired visual functions of RGCs in db/db but not db/m mice. These findings suggest that early stage visual function decline is reversible and furthermore, that oxidative stress could play a pivotal role in mediating neural degeneration in diabetic retinas.

Deteriorated visual functions observed within the diabetic retina can be attributed to a variety of factors that lead to the loss of specific classes of neurons or synapses, changes in ion conductance within neurons, or alterations in the metabolic pathways that allow adequate passage of ions across cell membranes. The present results show that the ON- and OFF-RGCs show different trends of RF size reduction (Figure 1). The OFF system has a consistent ∼15% reduction in RF size from first two time points (8–12 wk) but that the ON system shows no reduction in RF size at the first time point but a substantial shrinkage at the second time point. The neuromechanisms underlying this early stage OFF-RGCs RF size decline remains to be investigated. Nevertheless, by 20 wk both ON and OFF systems are similarly affected. On the other hand, although the RF size decline in db/db mice has not been described before, several lines of morphological evidence from different diabetic animal models reveal that ON-bipolar cells are first affected by diabetic injury [8], ON-RGCs have enlarged dendritic fields [23], and an increase in dendritic branching patterns is detected in ON type RGCs [24]. However, whether the enlarged dendritic field size can be correlated to the reduced RF size remains undetermined. Furthermore, whether diabetes directly affects RGCs, or whether the results are all indirectly attributable to affects on upstream neurons such as photoreceptors remains unknown. The present study shows that the reduced RF size in 20 wk db/db mice can be improved by in vitro application of SOD. Thus it is plausible that synaptic connections and/or upstream neurons are stressed but not interrupted in 20 wk db/db mice. Contrary to the RF size reduction described above, the present study shows that, in comparison with 8 wk db/db mice, LT (Figure 2E) of ON-RGCs was significantly elevated in 12 and 20 wk db/db mice while that of OFF-RGCs was not. It is possible that diabetic injury selectively affected the center-surround organization of RF of ON-RGCs. Indeed, a recent clinical study provides psychophysical evidence that the difference of Gaussian (DOG) RF model fits the experimental data well for the normal controls but does not fit the data obtained for the diabetic patients. This is attributed to the dysfunction of the lateral inhibition processes beyond photoreceptors [45]. The present study shows that the luminance threshold of ON-RGCs was significantly elevated (Figure 2). This is in agreement with clinical evidence that the rod system is dysfunctional during the early phase of DR [46], [47]. Interestingly, physiological recordings from rod driven ON-bipolar cells also show that the GABA signaling pathway is altered in diabetic retinas [22]. Taken together, it is plausible that the impaired ON pathway in diabetic retinas could be affected by abnormalities in neurotransmitter release, receptor sensitivity, or expression.

ROS are thought to play a significant role in the development of diabetic retinopathy. The first line of defense against ROS is a class of detoxifying enzymes that scavenge ROS. These include the SODs and catalases. To date, however, no direct evidence supports the idea that ROS generation may impair the visual function of individual RGCs of diabetic retinas. However, as shown in Figure 3 and supported by other studies [48], retinal and intracellular ROS levels elevate substantially between weeks 8 and 20 of db/db mice development, indicating that the retinal neurons are suffering from oxidative stress [48]. Oxidative stress causes long-lasting modifications to the excitatory amino acid transport systems in retinal neurons, which can be reversed by some antioxidants [49]. Indeed, several effective treatments for diabetic retinopathy were mediated by a mechanism that involved the elimination of oxidative stress in db/db mice [26], [32]. This present findings suggest that a similar mechanism for RGCs. As shown in Figure 5D, *in vitro* application of SOD significantly recuperated the contrast gain and restored the reduced RF size of ON- and OFF-RGCs in db/db mice, indicating that oxidative stress indeed plays a role in impaired visual functions in early diabetic retinopathy. Taken together, these findings also suggest that the visual function of both ON- and OFF-RGCs were reversible in 20 wk old db/db mice. However, the ON- and OFF-RGCs of db/m mice act differently in response to the SOD treatment. The contrast gain of ON-RGCs was not affected by the SOD application but the contrast gain of OFF-RGCs was drastically suppressed. On the other hand, we did not observe any significant changes in the average RF size of ON-RGCs in db/m mice after SOD application, whereas the RF size of OFF-RGCs was significantly increased. Based on data collected from the present study, we do not have enough evidence to comment on the preferential effect of SOD on OFF-RGCs of db/m mice.

## Materials and Methods

### Animals

Forty-one female C57BKS/J db/db mice (8 wk: 15; 12 wk: 10; and 20 wk: 20), and forty-one female age-matched heterozygous db/m littermates (8 wk: 11; 12wk: 10, and 20wk: 20) (Jackson Laboratory, Bar Harbor, Maine, for strain background information visit http://jaxmice.jax.org/strain/000642.html) were used in this study. Animals were housed in a 12-hour light-dark cycle and food and water were provided ad libitum. Animal blood glucose levels were measured with a blood glucose meter (Accu-Chek active, Roche, Mannheim, Germany) at 9:00 am on every Monday and just before the experiment. Animals used in this study did not receive insulin treatment. All experiments were performed in accordance with Peking University guidelines for animal research and the ARVO Statement for the Use of Animals in Ophthalmic and Vision Research.

### In vitro preparation

The retinal preparation has been described previously. Briefly, the animal was dark adapted for 40 minutes before enucleation. Under dim red light, the lens and vitreous were carefully removed with a pair of fine-forceps. The eyecup was flat mounted, sclera side down, directly on the bottom of a recording chamber and was superfused by oxygenated (95% O_2_/5% CO_2_) Ames medium (Sigma-Aldrich, St. Louis, MO) at a fixed rate (5 ml/min) at room temperature between 22 and 24°C. RGCs recorded at this temperature showed similar response profiles as those recorded in vivo [50].

### Superoxide dismutase application

SOD (S5395, Sigma Chemicals Santa Louise, MO) was dissolved in the oxygenated Ames medium (300 unit/mL) and perfused over the flat mount retina throughout the duration of the experiment. The recording started one hour after the retina was incubated in the medium and lasted for four hours.

### Visual stimulation

Computer generated visual stimulation paradigms have been described previously [51], [52]. Briefly, visual stimuli were generated by programming a graphics card (Matrox Millennium 3000), displayed on a 5″ CRT monitor (Kristel Corp., St. Charles, IL) and imaged with a first-surface mirror and lens (Edmond Scientific, Barrington, NJ) on the film plane of the microscope's camera port. Luminance level of the CRT was measured with a digital radiometer (S370 Radiometer, UDT Instruments, San Diego, CA). The maximum luminance of CRT was 183 cd/m**^2^**. The background luminance levels on CRT were close to 0.4 cd/m**^2^**. During this experiment, a Zeiss 40x water-immersion objective was used (Carl Zeiss., Thornwood, NY). With this objective, the maximum luminance was further attenuated to 0.69 cd/m**^2^** on the retinal surface. Based on the assumption that, at 500 nm, 1 cd/m**^2^** = 1 lumen/m**^2^** = 3.68×10**^3^** photons/µm**^2^**/s, the unattenuated total irradiance was found to be 2.54×10**^3^** photons/µm**^2^**/s and the background irradiance was 5.55 photons//µm**^2^**/s. This intensity was further reduced by using neutral density filters (Oriel Corp., Stratford, CT).

### Physiological recording and data analysis

Visual responses were recorded extracellularly using a glass microelectrode, amplified with an intracellular amplifier (IR283; Neurodata Inc., Delaware Water, PA), and digitized with a low-noise analog/digital interface (Digidata 1430; Axon Instrument, Inc., Forest City, CA). After visualization with a nuclear stain dye (Acridine orange, 0.002%, Sigma-Aldrich), cells that had RGA1 morphology were selected for recording [53]. The luminance threshold tests were conducted following previously published methods [51], [52]. Briefly, the receptive field (RF) was mapped with a 0.2° testing spot. An area-threshold test was then performed to determine which spot size evoked the maximum discharge. After 40 minutes of dark adaptation, the optimized spot size was selected for the increment luminance threshold (LT) test. The intensity of CRT background and visual stimulation was programmed and attenuated in order to sufficiently maintain the retinas in their dark-adapted state. A criterion response for threshold irradiance was obtained by gradually increasing the intensity levels of the testing stimuli; a firing rate of two spikes per second above the baseline rate was set as a threshold response. If none of the test stimuli produced exactly two spikes per second, the threshold was determined by linear interpolation. This difference also matched our subjective auditory criteria. For contrast sensitivity test, the contrasts of drifting sinusoidal gratings were defined by percent Michelson contrast: 100 * ((Imax – Imin)/(Imax + Imin)), where Imax and Imin were the peak and trough intensities (range, 0–100%). We applied conventional Fourier analysis techniques to plot post-stimulus time histograms (PSTH) and to determine the amplitude of response components at the frequency of stimulation (fundamental) and at the second harmonic. We also plotted response magnitude (spk/sec) versus contrast curves at eight spatial frequencies ranged from 0.02 to 0.33 c/deg and selected contrast response curves that were evoked by the optimum spatial frequency. Contrast response functions, were fit with a Michaelis-Menten function R (α)  = αRmax / (α + Rmax/G): where *R* is spike rate at contrast α minus baseline firing rate, *G* is contrast gain (impulses per second per percentage contrast), and *R*max is maximum spike rate minus baseline firing rate. At low contrasts, the spike rate *R* increases linearly with contrast α with a slope of *G*. Thus the slope G was applied to evaluate a cell's contrast sensitivity. In the present study, the slope of a contrast response curve was selected for grating contrasts that were below 60%. The acquired data were further analyzed off-line (pCLAMP9; Axon Corp., CA). Additional statistical analysis, including fast Fourier analysis and ANONA, was performed with Microsoft Excel (Microsoft Corp., Redmond, WA).

### Detection of ROS generation

Since dihydroethidium (DHE) fluorescence can be used to detect both intracellular and extracellular superoxide generations[54], the generation of retinal ROS was assessed by DHE (Invitrogen Molecular Probes, Eugene, OR). Briefly, fresh retinas were harvested and quickly frozen in liquid nitrogen for cryosection (Leica CM1950, Leica Microsystems Ltd, Wetzlar, Germany). The cryosections (10 µm) were washed with warm phosphate buffered saline (PBS) solution and then incubated with 5 µM DHE for 30 min at 37°C in PBS. DHE specifically reacted with intracellular and extracellular superoxide anions and was converted to the red fluorescent compound ethidium. The sections were examined and photographed by using an inverted fluorescent microscope equipped with a digital camera (Eclipex Ti-S, Nikon Instech Co., Tokyo, Japan) under identical exposure conditions, and the optical density of the staining in the Outer Nuclear Layer (ONL), Inner Nuclear Layer (INL), and ganglion cell layer (GCL) was measured in randomly selected retinal slide images. Five measurements were taken at 200 µm intervals on each image using a commercial software program (Photoshop CS5, Adobe Corp., San Jose, CA, USA).

### Statistical analysis

All data are expressed as mean±standard error of the mean (SEM). To analyze a specific visual function of RGCs, two-way ANOVA was performed for ON RGCs and then another for OFF RGCs, where one factor was the animal type (dm versus db) and another factor was duration of diabetes (8, 12, and 20 wk). Then appropriate post-hoc analysis was conducted to determine when diabetes had an effect on a specific visual function. If only one factor was involved, one-way ANOVA was conducted and followed by Tukey's multiple comparison test. P< 0.05 was considered statistically significant.
